# Tunable Fabry-Perot Interferometer Designed for Far-Infrared Wavelength by Utilizing Electromagnetic Force

**DOI:** 10.3390/s18082572

**Published:** 2018-08-06

**Authors:** Dong Geon Jung, Jun Yeop Lee, Jae Keon Kim, Daewoong Jung, Seong Ho Kong

**Affiliations:** 1 School of Electronics Engineering, College of IT Engineering, Kyungpook National University, Daegu 41566, Korea; max700128@hanmail.net (D.G.J.); leejy@kitech.re.kr (J.Y.L.); 2 Aircraft System Technology Group, Korea Institute of Industrial Technology (KITECH), Daegu 42994, Korea; kjg@kitech.re.kr; 3 Department of Sensor and Display Engineering, Kyungpook National University, Daegu 41566, Korea

**Keywords:** FPI, IR detector, optical filter

## Abstract

A tunable Fabry-Perot interferometer (TFPI)-type wavelength filter designed for the long-wavelength infrared (LWIR) region is fabricated using micro electro mechanical systems (MEMS) technology and the novel polydimethylsiloxane (PDMS) micro patterning technique. The structure of the proposed infrared sensor consists of a Fabry-Perot interferometer (FPI)-based optical filter and infrared (IR) detector. An amorphous Si-based thermal IR detector is located under the FPI-based optical filter to detect the IR-rays filtered by the FPI. The filtered IR wavelength is selected according to the air etalon gap between reflectors, which is defined by the thickness of the patterned PDMS. The 8 μm-thick PDMS pattern is fabricated on a 3 nm-thick Al layer used as a reflector. The air etalon gap is changed using the electromagnetic force between the permanent magnet and solenoid. The measured PDMS gap height is about 2 μm, ranging from 8 μm to 6 μm, with driving current varying from 0 mA to 600 mA, resulting in a tunable wavelength range of 4 μm. The 3-dB bandwidth (full width at half maximum, FWHM) of the proposed filter is 1.5 nm, while the Free Spectral Range (FSR) is 8 μm. Experimental results show that the proposed TFPI can detect a specific wavelength at the long LWIR region.

## 1. Introduction

Infrared spectrometers that can detect specific wavelengths in the infrared (IR) region are required for various applications, such as in automobiles, military, medical treatment, and analysis, and there is an increased demand for improvements in spectroscopic technology. Therefore, in recent years, various studies have focused on IR detection sensors using IR spectrometry [1,2,3]. Among them, some studies focus on the tunable Fabry-Perot interferometer (TFPI) to finely control the wavelength range while using optical filters [4,5,6].

TFPI optical filters offer controllable optical wavelength resolutions and are employed as core components in Wavelength Division Multiplexer (WDM) networks [7], different methods of spectroscopy [8,9], and sensor applications [10,11,12]. In particular, MEMS (Microelectromechanical systems)-based TFPI filters are widely studied, owing to their advantageous features, including miniaturization, batch processing, cost-effectiveness, and insensitive polarization working. Generally, TFPI filters mainly consist of two independent mirrors with an air etalon gap. Multiple instances of light interference occur when some light passes through the input mirror. As a result, the set of a particular wavelength is determined at the output stage. By varying the distance or the refractive index between the mirrors, the wavelength characteristics can be varied over a wide range of wavelengths, facilitating the development of a tunable optical filter. A common method to create TFPI filters is to fabricate two movable parallel mirrors [13,14,15,16]. In principle, the distance between the mirrors can be adjusted with high accuracy while maintaining other parameters related to the two mirrors.

Previously reported TFPI-type wavelength filters primarily used an electrostatic force, and thermally operate in the visible- and near-IR light regions. However, there are drawbacks, such as high voltage, a narrow wavelength region, and difficulty of fabrication for the mid- and far-IR wavelength regions [17,18]. Furthermore, because an electrostatic force is applied to the upper and lower mirrors to adjust the distance between them, there is a disadvantage in that the detection of IR light is difficult because of the noise and low resolving power that is caused by the deterioration of parallelism of mirrors [19]. To date, owing to the many difficulties that are associated with this field of research, there have been few studies on the fabrication of TFPI-type wavelength filters that can operate in the far-IR light region. Because the distance between the upper and lower mirrors must be at least 7 μm for it to operate in the far-IR light region, it is difficult to fabricate TFPI, which operates in the far-IR light region, while using electrostatic forces [20,21,22,23]. Other drawbacks include the difficulty that is involved in fabrication using semiconductor processes, and, when compared with the wavelength filters that operate at low wavelengths, a very high voltage is required when the distance between the upper and lower mirrors is increased [24,25]. Furthermore, there is another disadvantage in that the parallelism of mirrors is decreased because the bending of mirrors occurs owing to the electrostatic force that is applied between the two mirrors.

Previously, we reported previously a Fabry-Perot interferometer (FPI)-based IR spectrometer by developing a novel polydimethylsiloxane (PDMS) patterning technique. The PDMS served as supporting layer to provide an air gap, and its thickness was controlled by the fabrication process parameters (etching time or etcher concentration). Once the fabrication of the FPI was completed, the thickness of PDMS was fixed, giving a specific wavelength characteristic [20].

In this study, to make improvements while considering these drawbacks, a TFPI-type IR light sensor was fabricated using an electromagnetic force. The focus of this study is to develop a supporting material between the upper and lower mirrors, and to filter the wavelengths in the wide region of the far-IR light. To adjust the distance between the mirrors using stress, PDMS, which has a high elastic force, was selected as a supporting material between the upper and lower mirrors. Moreover, a novel PDMS pattern technique was developed, because patterns of PDMS with a high aspect ratio were first required to have high deformation by stress. Second, there were many drawbacks in conventional pattern techniques with respect to their application to this experiment. To apply stress between the upper and lower mirrors, they were fabricated in such a way that wide spectrum detection would be facilitated using the electromagnetic force that is present between an ultra-small solenoid and permanent magnet. For the reflective film that was employed as the mirror, a silicon wafer was structurally etched, and aluminum was deposited while using micro electro mechanical systems (MEMS) technology. The thickness of the deposited aluminum was adjusted, and by comparing the transmittances, a thickness of aluminum with an appropriate transmittance was used for the reflective film. To detect the infrared light, a bolometer-type IR light sensing method was chosen, which has high electrical resistance changes that result from a temperature increase due to the incidence of IR light; in this study, the widely used α-Si was chosen [26]. The fabricated TFPI-type wavelength filter can operate better in the far-IR region when compared to conventional filters, and, as a single device, it exhibited the characteristic of filtering wavelengths over a very large region. IR light having a certain wavelength, which penetrates the TFP wavelength filter, can be detected when it is incident on the bolometer-type IR detector [27,28].

## 2. Experimental Details

### 2.1. Fabrication of TFPI

**Figure 1:**
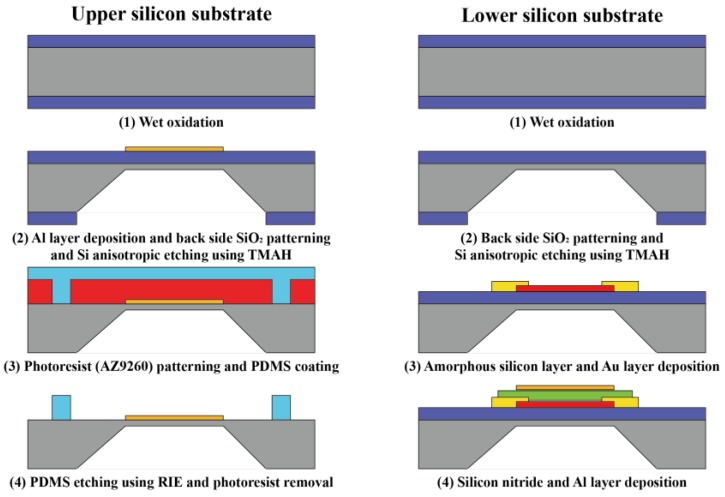
Tunable Fabry-Perot interferometer (TFPI) fabrication processes of upper and lower substrate.

The fabrication process of the proposed TFPI-based IR spectrometer is divided into two parts: the upper and lower substrates. Figure 1 shows the fabrication sequence of the proposed TFPI-based spectrometer’s upper and lower parts.

A p-type 6″-silicon wafer is used as the upper substrate. First, the wet oxidation process was conducted to grow an SiO_2_ layer on the Si wafer, and the SiO_2_ layer on the back side of the Si wafer was patterned by photolithography and a wet etching process, sequentially. The patterned SiO_2_ layer serves as mask layer in the Si wet anisotropic etching process while using a tetramethylammonium hydroxide (TMAH) solution. The thickness of the fabricated membrane is about 30 µm. Next, the aluminum (Al) layer was deposited on front side of the Si wafer for fabricating the upper reflector. Finally, a supporting column utilizing PDMS was fabricated on the front side of the Si wafer by photolithography and dry etching.

The lower part of the IR light sensor comprises a lower mirror and IR detector, and the IR detector is positioned under the lower substrate of the TFPI. Silicon oxide with a thickness of 1 μm, which will be used as an insulator film of the bolometer and substrate, is grown on the P-type 6″-Si substrate while using a 6″ furnace. α-Si, which is used for the IR light-detection part, is deposited using low-pressure chemical vapor deposition (LPCVD). The process is carried out at 580 °C and 250 mTorr atmospheric pressure, and after injecting SiH_4_ and PH_3_ in a 100:1 ratio, 500 Å α-Si is deposited. Subsequently, for α-Si, AZ5214 PR is patterned while using the photolithography process, and it is etched using SF_6_ gas with the reactive-ion etching (RIE) dry etching process. Then, the annealing process is conducted in nitrogen at 400 °C for 30 min. For the electrode that is used to measure the resistance of the bolometer’s sensing film, a 2000 Å thick layer of Au is deposited, and while using the lift-off process, patterning is conducted. α-Si has a characteristic whereby the resistance of the material changes according to the temperature change. Therefore, to increase the temperature of the α-Si sensing film by absorbing the IR light, a 2000 Å thick layer of Si_3_N_4_ is deposited on the α-Si at 200 °C for 12 min using plasma-enhanced chemical vapor deposition. With respect to the aluminum (Al) used as the lower mirror of TFPI, 30 Å was deposited to obtain a 30% transmittance. More detailed fabrication processes have been described in [20].

**Figure 2:**
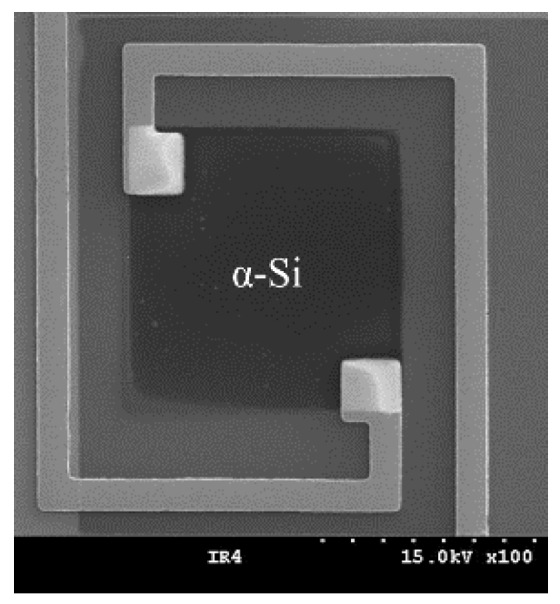
Microscope picture of fabricated bolometer.

Figure 2 shows the IR detector of the fabricated IR light sensor. α-Si was patterned in a square shape, and to minimize the heat loss, Au was deposited with a thin pattern. Then, by depositing Si_3_N_4_ and Au on α-Si to absorb the IR light, both the aluminum used as the lower mirror and α-Si were insulated. By doing this, the penetrated IR light of the lower mirror was absorbed in Si_3_N_4_, and because the temperature of α-Si was changed, owing to the temperature change of Si_3_N_4_, the resistance of α-Si was decreased and the IR light could be detected. Finally, by exposing the upper substrate to O_2_ gas and the plasma in the vacuum state for 30 s, the surface energy was increased, after which it was bonded to the lower substrate in the vacuum state.

### 2.2. PDMS Pattern Process and Design

Conventionally, pattern methods for PDMS are largely divided into three types: a PDMS pattern technique using photo PDMS, a PDMS pattern technique using a mold, and a PDMS pattern technique using RIE dry etching. However, conventional PDMS pattern techniques have several disadvantages in terms of their application to this experiment. Regarding the PDMS pattern technique while using photo PDMS, it is difficult to fabricate a pattern that has a high aspect ratio, and in this experiment, it is difficult to apply the PDMS pattern technique using a mold because a PDMS film is left behind, along with the desired pattern. Further, it is difficult to apply the PDMS pattern technique that uses RIE dry etching because of damage to the substrate after etching PDMS. Therefore, in this experiment, a PDMS pattern process is designed to fabricate clear PDMS patterns on a substrate without damaging it. This was carried out successfully through a photolithography process and dry etching [20]. To pattern PDMS on a 6″-Si substrate, 8.5-μm-high AZ9260 PR was patterned by performing a photolithography process. PDMS shows different elasticity values depending on the ratio of the subject and hardener. In this experiment, using Dow Corning Sylgard184, the main material and hardener are stirred in a 10:1 ratio, and the bubbles that are produced during stirring are removed using a vacuum pump for 30 min. After conducting the spin-coating for PDMS at 5000 rpm for 5 min, a PDMS film is formed on the photoresist. Next, the bubbles that were produced between the PDMS patterns and PDMS during the spin coating are completely removed by using the vacuum pump for 1 h. Then, hardening of the silicon wafer on which PDMS was spin coated, is conducted at 90 °C for 1 h. To form the PDMS patterns, only the PDMS film should be removed through dry etching.

**Figure 3:**
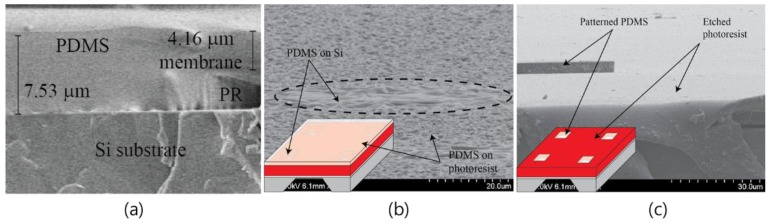
(**a**) Polydimethylsiloxane (PDMS) on Si substrate, (**b**) surface of PDMS membrane, and (**c**) dry etched Si substrate.

Figure 3a shows a cross-section of the PDMS film that was formed on the PR when designing the RIE dry etching process. To remove the hardened PDMS film, which has a thickness of 4.1 μm, dry etching was carried out using the RIE process. Figure 3b,c show the SEM images before and after the RIE dry etching process. When the dry etching was conducted for 12 min, 4.3-μm-thick PDMS was etched, and it was confirmed that there was a difference of about five times in the etching speeds of PDMS and AZ9260 PR. By etching the PDMS with a low etching rate to obtain uniform PDMS patterns with a height of 8 μm, damage to the Si substrate was prevented, and uniform PDMS patterns were fabricated.

**Figure 4:**
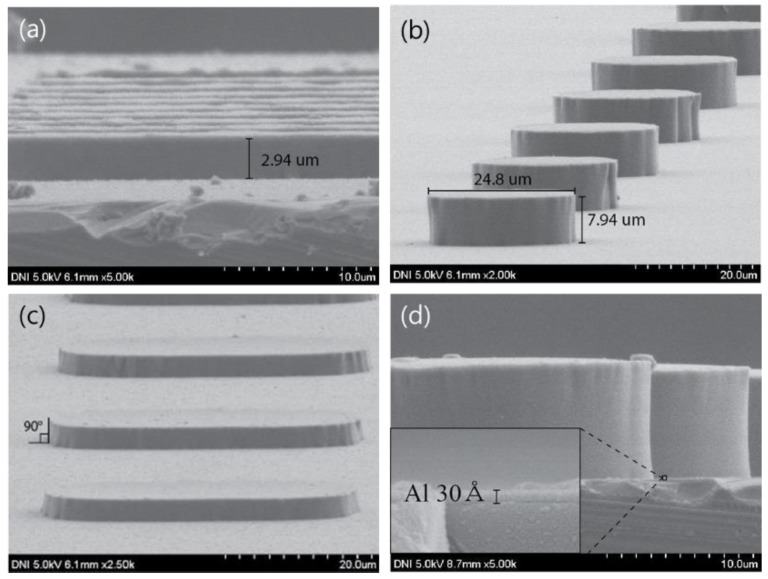
(**a**) PDMS on Si substrate, (**b**) surface of PDMS membrane, (**c**) and (**d**) dry etched PDMS on Al substrate.

Figure 4a shows an image of PDMS patterned on 30-Å aluminum. Figure 4b shows the inclination angles of the fabricated PDMS patterns. Figure 4c,d show the PDMS patterns fabricated with different heights. The PDMS pattern technique that is developed in this study can form a PDMS pattern that has a high aspect ratio because the inclination angle is almost 90°, and it can easily control the height of the PDMS pattern according to the photoresist type and the spin-coating speed.

Furthermore, because the AZ9260 photoresist that is used as the mold plays the role of a protection layer, there is an advantage in that the substrate will not be damaged; in addition, there is a benefit in that the residues of the substrate will not be left behind, thereby supplementing all of the drawbacks of the conventional PDMS pattern techniques.

### 2.3. Fabrication of Solenoid Structure

**Figure 5:**
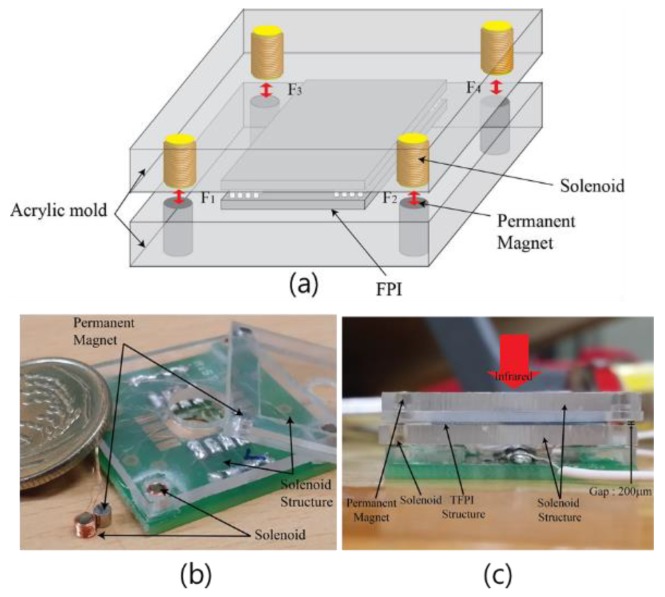
(**a**) Schematic, (**b**) top, and (**c**) cross-sectional view of the fabricated solenoid.

Figure 5 shows the solenoid structure that is used to apply stress on the TFPI structure using the electromagnetic force. The permanent magnet was fabricated in a cylindrical shape with a 2-mm diameter and 2-mm height. Aluminum, which is a paramagnetic material with a 1-mm diameter, was used as the core of the solenoid; depending on the 200 and 300 winding turns of the coil having a 50-μm thickness, the diameter of the solenoid is 2 mm and 2.6 mm, respectively, and the height is 2 mm [29]. In the lower solenoid structure, four solenoids were positioned and fixed at each corner. In the upper solenoid structure, the permanent magnets are fixed at the same positions of the solenoids. In this experiment, 720-μm-thick silicon wafers were used. Hence, by bonding two silicon wafers, a device with a thickness of about 1.4 mm was fabricated, with a 200-μm distance between the solenoid and thepermanent magnet. When the elements are combined, there is a 200-μm-thick gap between the solenoid and permanent magnet.

### 2.4. Measurement Method of TFPI-Type Infrared Detection Sensor

With respect to the measurements that were obtained using the TFPI-type IR light sensor that was fabricated in this study, measurements were performed separately with the IR light wavelength filter. For the IR light measurement of the wavelength filter part, a Fourier transform infrared spectroscopy (FT-IR) measurement device was used. By measuring the amount of penetrated IR light after illuminating an object with IR light, FT-IR can be used to find information about the blackbody inside the device, and then measure the transmittance, reflectance, and absorbance. Using the FT-IR measurement device, the IR light in the 2.5–18 μm wavelength region was scanned and measured. The wavelengths at which both destructive and constructive interference occurred were measured according to the incident IR light wavelength. More accurate measurements are possible in a vacuum state; however, because the electric current has to be applied on the device for the TFPI-type IR detection sensor, the measurement was carried out by removing the IR light absorbance in air using information that was related to the blackbody in air after taking measurements in the air state. For the measurement with the IR wavelength filter, the IR light-sensing film was not fabricated on the lower substrate, and after TMAH etching and 30-Å aluminum deposition, it was O_2_ plasma-treated and bonded with the upper substrate in the vacuum state; afterwards, the measurement was conducted separately while using the FPI filter.

## 3. Results and Discussion

### 3.1. Operation Principle

**Figure 6:**
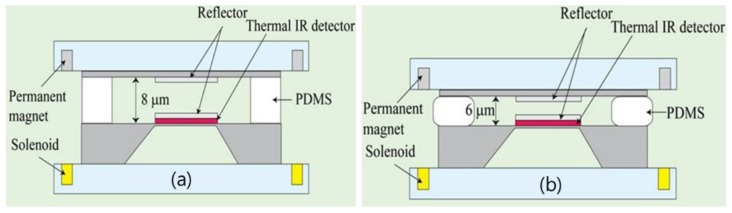
(**a**) Current-temperature characteristics of the micro heater and (**b**) temperature-resistance characteristics of the micro temperature sensor.

Figure 6 shows the operating principle of the fabricated device. The bolometer was fabricated under the lower reflector, so that when IR light with a specific wavelength that penetrated the FPI was incident on the bolometer, it was detected because of the change in the electrical resistance. To vary the distance between the reflectors, the solenoids and permanent magnets were fixed on the acryl mold, and by applying to the device a stress that was produced by the electromagnetic force, the distance between the reflectors was varied. By setting the distance between the reflectors as 8 μm, IR light with a wavelength of 16 μm was penetrated. Figure 6b shows the cross-section of the device in an electromagnetic force-applied state. Because of the stress that was present owing to the electromagnetic force between the solenoids and permanent magnets, the PDMS experienced stress from the outside and underwent deformation [30]. The TFPI was designed and fabricated in such a way that the filtered transmittance wavelength was adjusted and operated by adjusting the distance between the upper and lower mirrors, such that they are as high as the height of the strained PDMS patterns.

### 3.2. Designing IR Transmittance and Reflectivity

**Figure 7:**
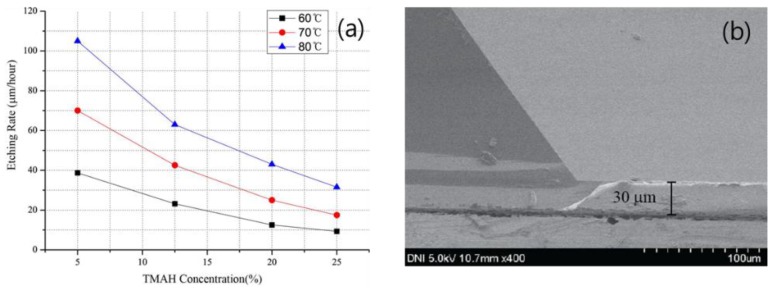
(**a**) Silicon etching rate as a function of tetramethylammonium hydroxide (TMAH) concentration with respect to temperature, and (**b**) etched silicon wafer using TMAH.

To minimize the IR light that was absorbed in the silicon substrates, a 30-μm-thick substrate was etched on the 740-μm-thick Si substrate. To perform etching on the Si substrate, SiO_2_ was patterned, and while using the TMAH solution, etching was conducted. The Si substrates having a (111) lattice structure were etched with an inclination of 54.7°. Figure 7a shows the variation in the etching rate, according to the TMAH concentration and temperature. Based on the measurement results, it was found that when the temperature of the TMAH solution was higher and the concentration was lower, the etching rate was faster. However, as the etching rate increased, the surface of the etched side became rougher with lower uniformity. Figure 7b shows the cross-section of the etched Si substrate. In this experiment, because the performance decreases as the surface of the Si substrate becomes rougher, a 710-μm thickness was etched slowly by heating the 25% concentration TMAH solution at 60 °C. Because etching is also conducted at a low rate on the SiO_2_ layer, which is used as a mask layer when etching the Si substrate while using TMAH solution, the surface of SiO_2_ becomes rough and its uniformity becomes low. In this experiment, because the uniformity of the Al that was used as a mirror was an important factor, the SiO_2_ that was grown on both sides of the Si substrate was removed using dilute hydro fluoric acid (DHF) solution. Because materials, such as Ag and Au, have close to 100% transmittance in the far-IR light region, Al having a transmittance of about 95% was chosen for the upper IR light reflector [20,28]. For FPI, the reflectivity of the reflector is one of the most important factors. When a material has a high reflectivity, the full width half maximum (FWHM) is narrow, thereby increasing the sharpness of the penetrating IR light, and thus, the FPI’s performance is improved. However, there is a disadvantage in that it is difficult to identify the noise because the transmittance decreases as the reflectivity increases. Therefore, in this study, to realize a design such that the IR light is incident on the bolometer with an appropriate transmittance, the transmittance of the Si substrate and Al was also considered in addition to the transmittance of Al itself. We previously confirmed the variation in the IR transmittance according to the thickness of aluminum [20]. Using the TMAH solution, the silicon substrate was etched 710 μm vertically; on the remaining 30-μm-thick Si substrate, the IR transmittance was measured while increasing the thickness of Al from 10 Å to 100 Å in steps of 10 Å. It was confirmed that as the thickness of Al increased, the transmittance decreased sharply, and at a thickness that is greater than or equal to 50 Å, almost no IR light penetrated. This experiment was conducted by depositing Al of thickness 30 Å having about 30% transmittance.

### 3.3. Performance Evaluation of TFPI-Type Infrared Light Sensor

**Figure 8:**
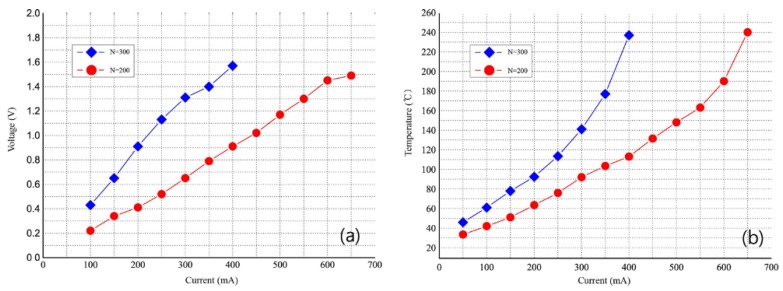
(**a**) Solenoid I-V characteristics and (**b**) temperature characteristics as a function of applied current, with different number of winding turns.

Figure 8a shows the voltage characteristics that were obtained by applying electric currents on the solenoids having different numbers of winding turns. When compared to the solenoid with 200 winding turns, the solenoid with 300 winding turns has about two times higher resistance. Figure 8b shows the temperature characteristics that were obtained by applying electric currents on the solenoids with different numbers of winding turns. The solenoids with 200 and 300 winding turns had a temperature of 200 °C at an electric current of 650 mA and 400 mA, respectively. At a temperature of 200 °C or higher, the permanent magnet loses its magnetic force owing to the decreased magnetic flux density. Therefore, in this experiment, a maximum current of 600 mA was applied by using the solenoid with 200 winding turns. Further, in this experiment, to minimize the errors of the bolometer that are caused by heat produced from the solenoids, the bolometer was positioned sufficiently far from the solenoids; however, in future, this can be achieved by heat-isolating the bolometer.

**Figure 9:**
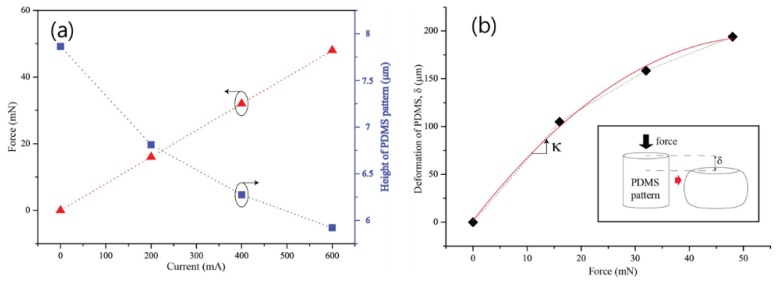
(**a**) Mechanical forces of solenoid and height of PDMS pattern as a function of current, and (**b**) deformation of PDMS as a function of the mechanical force of solenoid.

Figure 9a shows the variation in the mechanical force of the solenoid and the height of the PDMS pattern, according to the theoretical electric current. Theoretically, according to Ampere’s law, the internal magnetic field of the solenoid is defined as:

B = μ × (N/l) × I(1)

where μ is the transmittance of the solenoid core; the aluminum used as core has a transmittance of about 1. N denotes the number of winding turns, l is the length of the coil, and I is the applied current.

As the current increases, the electromagnetic force that is applied on the solenoid increases linearly. Because of the electromagnetic force between the solenoid and permanent magnet, the stress is applied on the PDMS pattern, and thus, the height varies according to the deformation of the PDMS pattern.

Figure 9b shows the height deformation of PDMS patterns according to the force that is applied between the solenoid and permanent magnet. The deformation was experimentally obtained from the measured spectra. As the force increases, it can be seen that the deformation of the PDMS pattern decreases non-linearly. Here, to determine the reciprocal of the slope of deformation of the PDMS pattern according to the force, the spring constant of the PDMS pattern is shown. In the linear section, the spring constant of the PDMS pattern was measured as about 163.6 m/N. Because the spring constant gradually decreases, it is expected that the degree of deformation of the PDMS pattern will be saturated at a certain amount of force.

**Figure 10:**
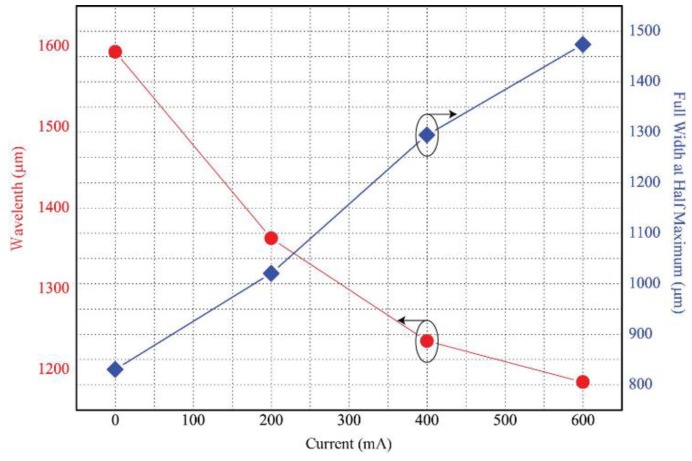
Peak transmittance wavelength shift and full width half maximum (FWHM) change as a function of applied current.

Figure 10 shows the variation in the peak wavelength and FWHM, according to the applied current. The FWHM indicates the width of the wavelength at a given position, which is half of the maximum and minimum values of the transmittance. The FWHM is one of the measures that determine the resolution of FPI, and as the FWHM becomes narrower, the resolution becomes higher. When the current of 600 mA was applied, a peak wavelength shift of about 4 μm was observed. This means that, owing to the electromagnetic force that is caused by the applied current, a deformation of about 2 μm occurred in the thickness of the PDMS pattern. In the case when a current of 200 mA was first applied, it could be seen that there was a shift in the wavelength with the largest width. As confirmed in Figure 10, because there was a greater deformation of the PDMS pattern owing to the greater stress, the wavelength shift decreased as the electric current increased.

**Figure 11:**
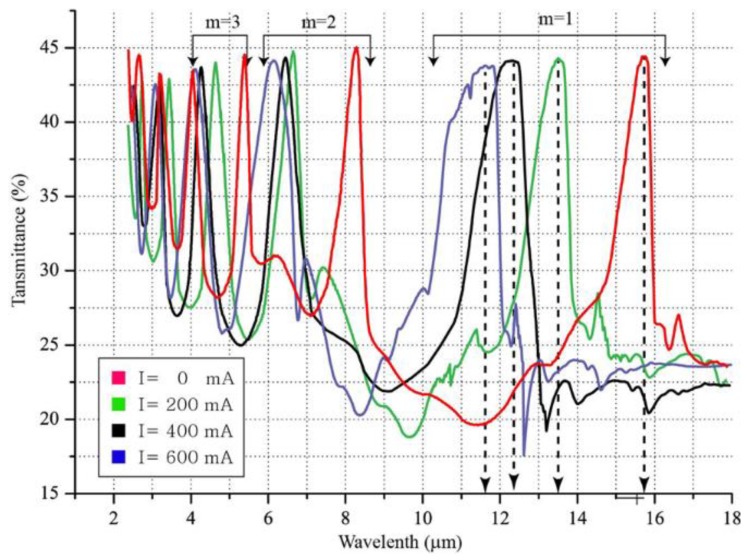
Comparison of infrared transmittance as a function of the applied current.

Figure 11 shows the IR transmittance as a function of the applied current in the state where the current is not applied to the solenoid. The optical performance of the TFPI is summarized in Table 1. When the order of the interference is 1 and the medium between the mirrors is air, theoretically, the peak IR transmittance wavelength should appear at the wavelength corresponding to twice the distance between the mirrors. In this study, by designing the PDMS pillar as 8 μm, the peak transmittance wavelength of IR light theoretically appears at 16 μm. In the case where no current was applied, the wavelength characteristics were seen with an error of about 100 nm between the actual measurement and theoretical value. When a current of 200 mA was applied on the solenoid, the IR light transmittance was constant, and compared with the case without the electric current, the peak transmittance wavelength shifted by about 2 μm. When currents of 400 mA and 600 mA were applied, it was confirmed that the peak transmittance wavelength shift decreased, and the peak transmittance wavelength shifted by 11.7 μm. By comparing the transmittance wavelengths of the IR light according to the currents that are applied on the solenoid, it was found that the peak wavelength shifted with an increase in the applied current. In TFPI, the free spectral range (FSR) should be considered, which is the distance of the peak transmittance wavelength between the orders of interference. That is, FSR corresponds to the wavelength region from 16 μm to about 8 μm, at which a peak wavelength appears when the order of interference is 1 and 2, respectively. Therefore, a shift of the peak wavelength according to the applied current is possible up to a maximum of 8 μm, where the wavelengths do not overlap. In the fabricated device, when the current of 600 mA was applied, there was an overlap in the peak transmittance wavelengths when the order of the interference was four. Therefore, the fabricated device facilitates IR detection of certain wavelengths only up to the order of an interference of three.

## 4. Conclusions

In this study, TFPI for far IR light was fabricated by applying the PDMS pattern technique. The conventional TFPIs were operated by producing an electrostatic force between mirrors, by applying current on the upper and lower mirrors to adjust the distance between them. However, because the distance between the mirrors is adjusted by applying an electromagnetic force on them, the mirrors have to be very thin, and thus, when the electromagnetic force is applied, the mirrors were bent, and the parallelism was deteriorated. Furthermore, to shift the wavelength, a very high voltage is needed, and there is a drawback in that fabrication is difficult in the long-wavelength region. To address this issue, in this study, a wavelength filter was fabricated to filter the IR light of the mid- and far-IR light regions; as compared to the conventional TFPI, there is an advantage in that there is a large shift in the spectrum.

Furthermore, a novel PDMS pattern technique was developed while using photolithography and RIE dry etching. The developed PDMS pattern technique facilitates the fabrication of patterns having high aspect ratios without causing any damage to the substrates. Furthermore, it has another advantage in that because PDMS can be patterned with a very low height, it can be applied to TFPI fabrication for not only the mid- and far-IR light regions, but also the visible-light region or near the IR light region. When a current of 600 mA was applied to the solenoid, the thickness of PDMS was changed from 8 μm to 6 μm, resulting in a peak wavelength shift of about 4 μm. A specific IR wavelength can be selected by adjusting the air etalon gap and the thermal IR detector senses the transmitted IR wavelength and converts it into electrical signal by changing the resistance of the thermal IR detector. The TFPI-type wavelength filter that realizes an improved performance and can operate in the far IR light region when compared to the conventional TFPI-type wavelength filter was developed and applied to IR light detection sensor. Because IR light in various wavelength regions can be detected using a single device, this technique has potential for application in various areas, such as automobiles, food, and military applications.

## Figures and Tables

**Table 1:** Summary of wavelength tuning performance.

Drive Current (mA)	0	200	400	600
Fabry-Perot gap (μm)	8	6.75	6.25	6
Peak wavelength (μm)	15.8	13.5	12.2	11.7
3-dB BW, FWHM (μm)	8.3	10.2	13	14.7
FSR (μm)	8	7	6	5

## References

[1] Rogalski A. (2011). Recent progress in infrared detector technology. Infrared Phys. Technol..

[2] Kong S.H., Wolffenbuttel R.F. (2005). Spectral Performance of a Micromachined Infrared Spectrum Analyzerin Silicon. IEEE Trans. Instrum. Meas..

[3] Chen C.N. (2012). Fully quantitative characterization of CMOS–MEMS polysilicon/titanium thermopile infrared sensors. Sens. Actuators B Chem..

[4] Banks J.M., Flammer P.D., Furtak T.E., Hollingsworth R.E., Collins R.T. (2012). Plasmonic Band-Pass Microfilters for LWIR Absorption Spectroscopy. Int. J. Opt..

[5] Takahashi K., Oyama H., Misawa N., Okumura K., Ishida M., Sawada K. (2013). Surface stress sensor using MEMS-based Fabry–Perot interferometer for label-free biosensing. Sens. Actuators B Chem..

[6] Enomoto T., Suzuki M., Iwaki T., Wado H., Takeuchi Y. (2013). Infrared absorption sensor for multiple gas sensing. Development of a Fabry-Perot spectrometer with ultrawide wavelength range. Electron. Commun. Jpn..

[7] Aziz M., Pfeiffer J., Wohlfarth M., Luber C., Wu S., Meissner P. (2000). A new and simple concept of tunable two-chip microcavities for filter applications in WDM systems. IEEE Photon. Technol. Lett..

[8] Li S., Zhong S., Xu J., He F., Wu Y. (2012). Fabrication and characterization of a thermal tunable bulk-micromachined optical filter. Sens. Actuators A Phys..

[9] Lammel G., Schweizer S., Schiesser S., Renaud P. (2002). Tunable optical filter of porous silicon as key component for a MEMS spectrometer. J. Microelectromech. Syst..

[10] Musca C.A., Antoszewski J., Winchester K.J., Keating A.J., Nguyen T., Silva K.K.M.B.D., Dell J.M., Faraone L., Mitra P., Beck J.D. (2005). Monolithic integration of an infrared photon detector with a MEMS-based tunable filter. IEEE Electron Device Lett..

[11] Lee C.L., Liu K.W., Luo H.H., Wu M.S., Ma C.T. (2017). A hot-polymer fiber fabry-perot interferometer anemometer for sensing airflow. Sensors.

[12] Chen Z., Xiong S., Gao S., Zhang H., Wan L., Huang X., Huang B., Feng Y., Liu W., Li Z. (2018). High-temperature sensor based on fabry-perot interferometer in microfiber tip. Sensors.

[13] Calaza C., Fonseca L., Moreno M., Marco S., Cané C., Gracia I. (2004). A surface micromachining process for the development of a medium-infrared tuneable Fabry–Perot interferometer. Sens. Actuators A Phys..

[14] Yamanoi T., Endo T., Toshiyoshi H. (2008). A hybid-assembled MEMS Fabry-perot wavelength tunable filter. Sens. Actuators A Phys..

[15] Parashar A., Shah A., Packirisamy M., Sivakumar N. (2007). Three cavity tunable MEMS fary perot interferometer. Sensors.

[16] Jeong J.W., Jung I.W., Jung H.J., Baney D.M., Solgaard O. (2010). Multifunctional tunable optical filter using MEMS spatial light modulator. J. Microelectromech. Syst..

[17] Tayebati P., Wang P.D., Vakhshoori D., Sacks R.N. (2002). Widely tunable fabry–perot filter using Ga(Al)As–AlO deformable mirrors. IEEE Photonics Technol. Lett..

[18] Russin T.J., Kerber M., Russin A., Wang A., Waters R. (2012). Fabrication and analysis of a MEMS NIR Fabry-Perot Interferometer. J. Microelectromech. Syst..

[19] Fernandez C., Guenther B.D., Gehm M.E., Brady D.J., Sullivan M.E. (2007). Longwave infrared (LWIR) coded aperture dispersive spectrometer. Opt. Express.

[20] Jung D.G., Kim H.R., Back S.M., Bang S.J., Kong S.H. (2015). A fabry-perot interferometer-based long-wavelength infrared spectrometer utilizing an novel PDMS patterning technique. Jpn. J. Appl. Phys..

[21] Batic M., Burger J., Cindro V., Kramberger G., Mandic I., Mikuz M., Stude A., Zavrtanik M. (2011). Verification of High Dose Rate 192 Ir Source Position During Brachytherapy Treatment Using Silicon Pixel Detectors. IEEE Trans. Nucl. Sci..

[22] Vargas-Rodriguez E., Rutt H.N. (2009). Design of CO, CO_2_ and CH_4_ gas sensors based on correlation spectroscopy using a Fabry–Perot interferometer. Sens. Actuators B Chem..

[23] Han S.W., Neikirk D.P. (2005). Design of infrared wavelength-selective microbolometers using planar multimode detectors. Proc. SPIE.

[24] Colace L., Sorinello V., Balbi M., Assanto G. (2007). Germanium near infrared detector in silicon on insulator. Appl. Phys. Lett..

[25] Wang J., Chen X., Hu W., Wang L., Lu W., Xu F., Zhao J., Shi Y., Ji R. (2011). Amorphous HgCdTe infrared photoconductive detector with high detectivity above 200 K. Appl. Phys. Lett..

[26] Choi J.C., Lee J.K., Choi Y.C., Jung D.G., Kong S.H. (2013). Infrared detector array with an incident-ray concentrator. Jpn. J. Appl. Phys..

[27] Chan K.L., Ning Z., Westerdahl D., Wong K.C., Sun Y.W., Hartl A., Wenig M.O. (2014). Dispersive infrared spectroscopy measurements of atmospheric CO_2_ using a Fabry–Pérot interferometer sensor. Sci. Total Environ..

[28] Yang J.C., Jung H., Lee G.J., Kang J.Y., Koo J.G., Park J.M., Park K.S., Kong S.H. (2008). Micro-Electro-Mechanical-Systems-based infrared spectrometer composed of multi-slit grating and bolometer array. Jpn. J. Appl. Phys..

[29] Lee J.K., Park K.W., Choi J.C., Kim H.R., Kong S.H. (2012). Design and fabrication of PMMA-micromachined fluid lens based on electromagnetic actuation on PMMA–PDMS bonded membrane. J. Micromech. Microeng..

[30] Balakrisnan B., Pati S., Smela E. (2009). Patterning PDMS using acombination ofwetand dry etching. J. Micromech. Microeng..

